# Presence of regulatory T-cells in endometrial cancer predicts poorer overall survival and promotes progression of tumor cells

**DOI:** 10.1007/s13402-022-00708-2

**Published:** 2022-09-13

**Authors:** Thomas Kolben, Mareike Mannewitz, Carolin Perleberg, Konstantin Schnell, David Anz, Laura Hahn, Sarah Meister, Elisa Schmoeckel, Alexander Burges, Bastian Czogalla, Anna Hester, Sven Mahner, Mirjana Kessler, Udo Jeschke, Stefanie Corradini, Fabian Trillsch, Susanne Beyer

**Affiliations:** 1 Department of Obstetrics and Gynecology, University Hospital, LMU Munich, Marchioninistr. 15, 81377 Munich, Germany; 2 Center of Integrated Protein Science Munich, Division of Clinical Pharmacology, University Hospital, LMU Munich, Munich, Germany; 3 Institute of Pathology, University Hospital, LMU Munich, Munich, Germany; 4 Department of Obstetrics and Gynecology, University Hospital Augsburg, Augsburg, Germany; 5 Department of Radiation-Oncology, University Hospital, LMU Munich, Munich, Germany

**Keywords:** Endometrial cancer, Regulatory T-cells, FoxP3, Immune escape, Survival

## Abstract

**Purpose:**

Endometrial cancer (EC) is one of the most common gynaecologic malignancies. Tumor infiltrating regulatory T-cells (Treg) have been reported to have a prognostic impact in many malignancies. Immunotherapeutic strategies are gaining interest for advanced and recurrent EC cases, where treatment options are rare. Our study was aimed at determining the value of Treg in EC progression.

**Methods:**

EC specimens from 275 patients and 28 controls were screened immunohistochemically for the presence of Treg represented by FoxP3. Correlations with clinicopathological and survival parameters were performed. Functional assays were performed using EC cell lines Ishikawa + and RL95-2 after co-culturing with isolated CD4 + CD25 + CD127dim Treg. To assess the influence of EC on the composition of peripheral blood mononuclear cells (PBMC), flow cytometric analyses were performed.

**Results:**

We found that an increased infiltration of Treg was associated with high grades and a reduced overall survival. Treg were almost absent in endometrium tissues from healthy control patients. Co-culture of tumor cells with CD4 + CD25 + CD127dim Treg led to functional changes: enhanced invasion, migration and viability indicated that increased levels of Treg in the tumor microenvironment may promote tumor growth. Furthermore, we found that the presence of EC cells led to phenotypic changes in PBMC, showing significantly increased levels of CD25 and FoxP3.

**Conclusion:**

Our results indicate that the presence of Treg in the EC tumor environment is associated with a poorer outcome. A remarkable impact of Treg on tumor cell behaviour and vice versa of tumor cells on PBMC subpopulations support this notion mechanistically. Our findings provide a basis for focusing on Treg as potential future therapeutic targets in EC.

**Supplementary Information:**

The online version contains supplementary material available at 10.1007/s13402-022-00708-2.

## Introduction

Endometrial carcinoma (EC) is the sixth most common cancer among women worldwide [1] and even the fourth most common malignancy in developed countries with an increasing incidence [2]. The *International Agency for Research and Cancer* estimated an incidence of 382.069 cases and a mortality of almost 90.000 worldwide in 2018 [1]. An imbalance in the estrogen level is one of the main risk factors for developing EC. Further risk factors include obesity, diabetes mellitus, nullipara, early menarche or therapy with tamoxifen [3]. EC was classified historically in Type I (estrogen-dependent) and Type II (estrogen-independent) by Bokhman [4]. Based on these criteria therapeutic procedures comprising surgery, radiotherapy and chemotherapy can be adapted dependent on the individual risk.

15% of EC patients suffer from recurrences and especially in these cases, therapeutic options are limited [5]. Beside FIGO-stage and grading, immune-related genes have also been identified to predict the prognosis of EC patients [6, 7]. Based on these findings, new therapeutic strategies were developed, including modulation of the immune system by the PD-1 inhibitor Pembrolizumab [8]. Patients with high microsatellite instability exhibit higher immune cell infiltrations in tumor tissues and better response rates to immunotherapy [9]. Immune cells are part of the tumor microenvironment (TME), which also includes mesenchymal cells, extracellular matrix and inflammatory mediators [9]. Interactions between tumor cells and the TME influence tumor growth, metastasis and survival [10]. Regulatory T-cells (Treg) are an important component of the TME [11]. This subtype of CD4 + CD25 + lymphocytes, discovered by Sakaguchi [12], suppresses the proliferation of effector T-cells and pro-inflammatory cytokine production [13, 14]. In healthy individuals, they play an important role in the prevention of autoimmune diseases [15] by inhibiting peripheral auto-immune-responses [16]. In cancer, however, Treg in the TME lead to immune escape and a worse survival rate [14, 17].

The transcription factor Forkhead box protein P3 (FoxP3) is essential for the development of Treg [18] and is their most specific marker, especially in combination with CD4 and CD25 [19–21]. Nevertheless, there is a need for further research in this regard, as a plasticity in Treg phenotype has been observed, giving rise to strict subgroups with Treg unique functional properties ranging from highly immune suppressive to immune reactive. For example, the subpopulation with the strongest immunosuppressive potential has been identified by absence of CD45RA, high CCR4 and other markers in addition to CD25 and high FoxP3 [22–24]. In some tumor entities FoxP3 has also been described as being expressed by the tumor cells themselves [25–27], but the existing literature is not conclusive here. In contrast, the impact of FoxP3 + Treg has been confirmed in many studies, although Guo et al. failed to find a convincing association with overall survival (OS) or recurrence free survival (RFS) in EC [28]. The authors of this meta-analysis, performed in 2020, suggested further studies in order to clarify the role of FoxP3 in EC.

In this study, we examined the effect of Treg, marked by FoxP3, in EC and its tumor microenvironment. We co-cultured EC cells with Treg to examine their effect on tumor cell viability, invasion, migration, proliferation and apoptosis. Additionally, shifts in the phenotype of PBMC were investigated under the influence of tumor cells.

## Materials and methods

### Patients and specimens

EC specimen of 275 patients, who underwent surgery in the Department of Gynecology and Obstetrics of the Ludwig-Maximilians-University of Munich from 1990 to 2002 were obtained and included. The present study was approved by the local ethics committee of the Ludwig-Maximilians-University of Munich (reference number 19–249). Due to the typically low number of non-endometrial histological subtypes, only patients with endometrial adenocarcinoma were included in our study. Clinicopathological and survival data were provided by the Munich cancer register. Histological subtype and grading were confirmed by the Department of Pathology, Ludwig-Maximilians-University Munich. The revision of the International Federation of Gynecology and Obstetrics (FIGO) system from 2009 was respected and applied to the whole collective [29]. The grading of endometroid carcinomas, complementary to FIGO staging, was based on the proportion of solid areas and the degree of differentiation. With increased grading, the proportion of solid tumor growth increased and the cells were increasingly poorly differentiated. [30]. A control group of 28 age-matched endometrium specimens was established. For this, patients who underwent surgery because of descensus uteri, uterine fibroids or clarification of postmenopausal bleeding were enrolled. Malignant and inflammatory processes were excluded in each case according to medical records. The control specimens were collected routinely between 2000 and 2002. All patients’ data were anonymized and patients have given informed consent before their tissue was stored. The ethical principles adopted in the Declaration of Helsinki 1975 have been respected.

### Immunohistochemistry

Paraffin-embedded tissue microarrays (TMA, surface 0.785 mm^2^ each spot) of 275 patients with EC were stained with a mouse anti-FoxP3 antibody (Dilution 1:300; ab 20,034, Abcam, Cambridge, UK) using ZytoChem Plus HRP Polymer System mouse/rabbit (Zytomed, Berlin, Germany) in accordance with the manufacturer’s instructions. 3,3-diaminobenzidine (DAKO DAB + ; Agilent technologies, Santa Clara, CA, USA) was used as chromogen. Positive and negative control staining were performed initially (Supplement 1). FoxP3 positive cells were counted in each spot with a light microscope (lens 20x, Leitz Diaplan lighmicroscope (Leica Microsystems, Wetzlar, Germany)) and the mean of three spots was calculated for each patient. In addition, the ratio of tumor cells to stromal cells was determined for each spot and the number of FoxP3 positive cells was normalized to the ratio to ensure sufficient proximity of the evaluated tissue. The control group was stained according to the same protocol. Three representative fields of view of each sample were selected (equals an area of 0.503 mm^2^) and FoxP3 positive cells were counted. A normalisation to the evaluated area was performed to guarantee comparability between the TMA samples and the control group samples.

### Immunofluorescence

In order to characterize the FoxP3-expressing cells, double immunofluorescence staining was performed. 5 specimens of EC-patients were incubated with a mouse anti-FoxP3 antibody (Dilution 1:50; 236AIE7, Thermo Fisher Scientific, Waltham, MA, USA) and a rabbit anti-CD3 antibody (ready to use; N1580, DAKO, Agilent technologies, Santa Clara, CA, USA) or a rabbit anti-CCR4 antibody (Dilution 1:50; HPA031613, Atlas Antibodies, Bromma, SWE) after blocking with Ultra-Vision-Proteinblock (Thermo Fisher Scientific, Waltham, MA, USA). Goat-anti-mouse-Alexa-Fluor488- and Goat-anti-rabbit-Cy-3-conjugated antibodies (both Dianova, Hamburg, Germany) were used as secondary antibodies. Samples were fixed with Vectashield® H1200 mounting medium with DAPI (VectorLab, Burlingame, CA, USA) and analysed using an Axiophot fluorescent photomicroscope (Zeiss, Oberkochen, Germany) and AxioVision 4.8.1 Software.

### Cell lines and culture conditions

The human EC cell line RL95-2 was purchased from the American Type Culture Collection (ATCC, Manassas, VA, USA) and Ishikawa + ER cells were provided by the European Collection of Cell Cultures (ECACC, Porton Down, Salisbury, UK). Both were routinely cultured in RPMI-1640 medium + GlutaMAX (Gibco Life technologies, Carlsbad, CA, USA) and 10% fetal calf serum (Thermo Fischer Scientific, Waltham, MA, USA).

### Isolation of human PBMC and regulatory T-cells

Human PBMC were purified from healthy blood donors by density gradient centrifugation at 2000 rpm for 20 min with Biocoll Separating Solution (Biochrom, Darmstadt, Germany). Erythrolysis was performed after which the cell suspension was passed through a 0.45 mm filter (BD, Franklin Lakes, NJ, USA). Human Treg (CD4 + CD25 + CD127dim/- cells) were obtained from freshly isolated PBMC by magnetic-activated cell sorting (MACS) using a CD4 + CD25 + CD127dim/- Regulatory T-cell Isolation Kit II (Miltenyi Biotec, Bergisch Gladbach, Germany) according to the manufacturer`s protocol in a two-step isolation. Firstly, non-CD4 + and CD127high cells were removed by negative magnetic selection and, secondly, CD25 + cells were collected using CD25 positive selection magnetic beads. Purity of the isolation was confirmed by flow cytometry and found to be around at 75% SD (Supplement 2). Next, the cells were labelled with CD4-PE, CD127-APC, CD25-FITC, CD19-PacBlue and CD3-APC (all BioLegend, San Diego, CA, USA). Intracellular staining for FoxP3 was performed using a PacBlue anti-human FoxP3 Antibody and FoxP3 Fix/Perm Buffer Set (BioLegend, San Diego, CA, USA) following the manufacturer’s protocol. To check the quality of the isolation, a Zombie NIR Fixable Viability Kit (BioLegend, San Diego, CA, USA) was added once after which a viability over 90% was found in each isolation fraction.

### Co-culture of tumor cells and immune cells

For cell culture experiments, 1 × 10^5^ tumor cells (Ishikawa + or RL95-2) per well were transferred to a 24-well plate. 1 × 10^6^ PBMC or 1 × 10^4^ Treg were added either in a 0.4-μm-pore Costar Transwell insert (Corning Incorporated, Kennebunk, ME, USA) or directly to the tumor cells without a physical separation. Co-culture was performed at 37 °C for 72 h. PBMC or tumor cells were harvested separately by taking advantage of their different adherence behaviour and washed with PBS for further experiments. Ishikawa + , RL95-2 or PBMC without previous co-culture were chosen as control in each experiment.

### Flowcytometry of PBMC

PBMC were labelled as described above after co-culture. PBMC of four different blood donors were co-cultured separately and one to three technical replicates were performed based on the limited availability of cells. Cells were detected using a BD LSRFortessa flow cytometer (BD, Franklin Lakes, USA). To analyse the prevalence of Treg within the PBMC populations, CD25 + FoxP3 + Treg were expressed as a percentage of total CD4 + T-cells. CD127 gating was additionally performed to confirm the gating strategy. The gating strategy and analysis of PBMC-viability is presented in Supplement 3. Two additional biological replicates were analysed by expanding the staining strategy by CD45RA-APC/Cy7 (BioLegend, San Diego, CA, USA). Flow cytometric analysis was performed using FlowJo 10.7.1 (BD, Franklin Lakes, USA). Since the PBMC and Treg originated from different blood donors, individual variability is anticipated and, therefore, figures were constructed depicting fold changes to controls for the following flow cytometric analyses and cell assays.

### Real time qPCR of sorted CD4 + lymphocytes

After co-culture with tumor cells, sorting of PBMC was performed using FACSAria III. The obtained CD4 + cells were lysed for RNA-Isolation (RNeasy Mini Kit, Qiagen, Venlo, Netherlands) and, subsequently, the RNA was reverse transcribed to cDNA (Biozym cDNA Synthesis Kit, Oldendorf, Germany). Real time qPCR (RT-qPCR) was performed by Applied Biosystems 7500 Fast Real.Time PCR using a TaqMan Fast Universal PCR Master Mix and Taqman Gene Expression Assay (all from Thermo Fisher Scientific, Waltham, MA, USA). Relative expression of the target genes IFNγ, TGFβ and IL10 to the housekeeping gene β-Actin was calculated using the 2^−ΔΔCt^ formula.

### Transwell invasion assay

The invasive ability of the tumor cells was assessed using a Transwell assay. A 8 μm-pore pore Falcon permeable support chamber (Corning Inc., Kennebunk, ME, USA) was covered with Matrigel (Corning Inc., Kennebunk, ME, USA) diluted 1:30 with sterile medium. Next, 2 × 10^5^ cells/well were seeded in the upper chamber and cultured in a 5% CO2 incubator at 37˚C for 48 h. Subsequently, the cell coating was removed from the upper chamber with a cotton swab. The cells that invaded to the reverse side of the membrane were fixed with 4% formaldehyde and methanol and stained with Mayer`s hemalum. The membranes were cut out and evaluated (20 × objective). Four fields of view were randomly selected. The cells were counted in each field of view after which the mean was calculated. The experiments were performed in biological triplicates.

### Scratch wound healing assay

5 × 10^5^ RL-95–2 and 2 × 10^5^ Ishikawa + cells were seeded into 24-well plates and incubated overnight to generate monolayers. A 100 μl pipette tip was used to create lesions (‘wounds’) after which images were taken after 0, 24, 48 and 72 h using an inverse phase contrast microscope (Leica Dmi1; Leica, Wetzlar, Germany) equipped with a LEICA MC120 HD camera (Leica, Wetzlar, Germany). Medium was changed every day before image acquisition. Data were analysed using ImageJ 1.52e (NIH, Bethesda, MD, USA) and the wound closure areas were normalised to the control group without previous incubation with Treg. The experiments were validated three times (*n* = 3).

### Caspase-3 apoptosis assay

To analyse apoptosis of RL95-2 and Ishikawa + cells, a Caspase-3- ELISA kit (R&D Biotechne, Minneapolis; MN, USA) was used according to the manufacturer’s instructions. 5 × 10^5^ cells were collected for each group. Biological triplicates and technical duplicates were performed.

### Cell viability assay

RL-95–2 and Ishikawa + cells, harvested after co-culture with Treg, were seeded in 96-well plates (2 × 10^4^ cells/well). After 4 h, cells were adherent and 3-(4,5-dimethylthiazol-2-yl)-2,5-diphenyltetrazolium bromide (MTT) was added to the medium. The reaction was stopped by adding 1 M sulfuric acid after which absorbance was measured at 595 nm using an ELx800NB microplate reader and Gen5 software (both BioTek Instruments, Winooski, VT, USA). Every experiment was repeated three times and in technical triplicates for each.

### Cell proliferation assay

Analogue to the MTT assay, 1 × 10^4^ RL 95–2 or Ishikawa + cells were seeded in 96-well plates and cultured for 4 h. Next, a 5-bromo-2-deoxyuridine (BrdU) incorporation assay (Roche, Basel, Suisse) was performed in accordance with the manufacturer’s protocol. After twenty hours of incubation with BrdU, a microplate reader was used to measure the optical density at 450 nm. Three biological and three technical replicates were performed. Proliferation was normalized to the proliferation rate of the control group.

### Statistics

Statistical analyses and data processing were performed using Excel and SPSS 26.0 (SPSS, Inc., Chicago, IL, USA). Survival analyses were performed by log-rank-test based on Kaplan–Meier curves. The median number of FoxP3-positive cells per spot was chosen as cut-off to generate equally distributed and thus comparable groups. Cox regression analysis was used for multivariate analyses to predict overall survival (OS) from the number of FoxP3 + cells. Stepwise increasing numbers of covariates (age, grading, FIGO, pT-stage) were included in order to map the independent association between FoxP3 and OS. In the course of this, FIGO III and IV, as well as pT 3 and 4 were merged, since only few patients had FIGO IV/ pT 4. Bivariate correlations between FoxP3 and clinicopathological variables were calculated by Spearman’s-rank-correlation coefficient. Differences among groups in histological analysis as well as cell culture settings were compared using Kruskal–Wallis-test/Mann–Whitney-U-test (2-sided asymptotic significance). Differences were considered to be statistically significant at *p* < 0.05. Significances are indicated by asterisks as follows: *: *p* < 0.05, **: *p* < 0.01, ***: *p* < 0.001.

## Results

### Patient characteristics

The clinicopathological characteristics of the analysed EC patients are depicted in Table 1. The majority of patients was diagnosed with FIGO I stage (77.1%) and low Grading G1 (58.9%). The mean age at the time of diagnosis was 64.6 years (median 64.8 years, range 35–87 years). The maximum follow-up reported by the cancer register was 318 months. Considering the fact that the median age of the collective is quite high, as is characteristic for EC, we limited the follow-up time to 200 months. After this time, death without connection to the disease is considered probable. Median overall survival in this time period was 138.1 months and 128.6 months for progression-free survival (PFS). 61.1% of the patients received only surgical therapy, 34.2% received surgery and radiation therapy. The remaining 4.8% received different therapy combinations including chemotherapy, radiation therapy and hormonal therapy.

**Table 1:** Demographic and clinical characteristics of the study population

Characteristics	Patient no (*n* = 275)	%
Age at diagnosis (years)		
< 65 > 65	140 135	50.9 49.1
Tumor size pT		
pT1 pT2 pT3 pT4 not available	220 18 32 3 2	80.0 6.5 11.6 1.1 0.7
FIGO staging		
I II III IV not available	212 17 38 6 2	77.1 6.2 13.8 2.2 0.7
Grading		
G1 G2 G3 Not available	162 89 24 0	58.9 32.4 8.7 0.0
Nodal status		
pN0 pN1 pNX	176 21 78	64.0 7.6 28.4
Metastases		
pM0 pM1 pMX	138 5 132	50.2 1.8 48.0
Survival		
Alive Dead Not available	154 121 0	56.0 44.0 0.0
Progression		
None At least one Not available	226 49 0	82.2 17.8 0.0

### Tumor associated stroma-located FoxP3 expression in EC specimens and association with clinicopathological parameters

**Fig. 1:**
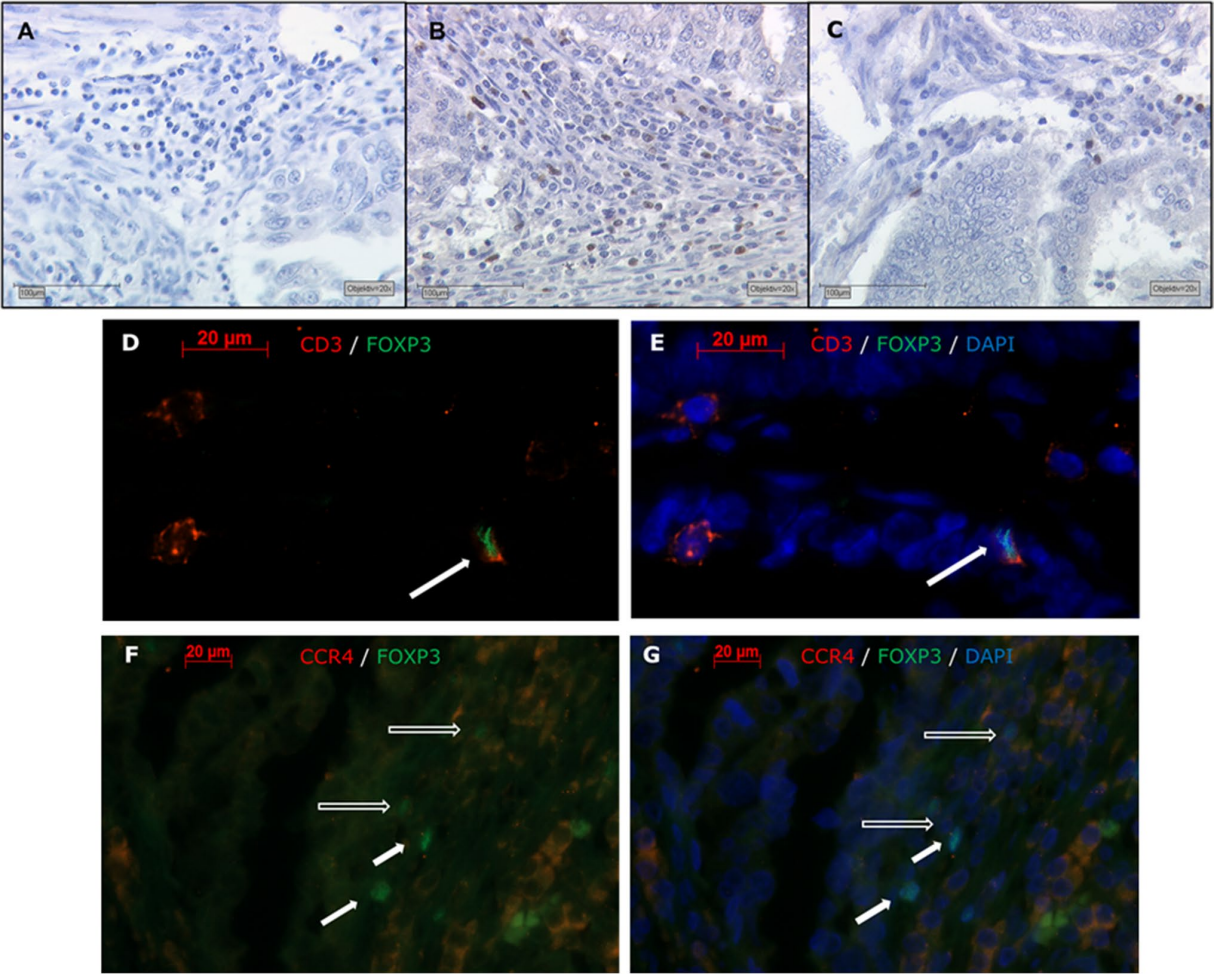
Nuclear expression of FoxP3+ lymphocytic cells in EC visualized by immunohistochemistry and immunofluorescene. Representative immunohistochemistry images of low/negative (**A**), high (**B**) and median (C) (*n* = 25,5/mm^2^). Treg cell density detected by FoxP3 staining (Scale bar 100μm, lens x20)

Immunohistochemical staining of nuclear FoxP3 revealed a positive expression in a total of 94.2% of all EC specimens (Fig. 1A-C). The median number of positive cells per spot was 25.5. The range was from 0.0 to 393.9 positive cells per spot, in 81.7% less than 100 cells per spot were counted. Nuclear FoxP3 was expressed exclusively by stroma infiltrating lymphocytes, which migrated between the tumor cells in some cases. Shape and size of the positive cells led to the assumption of being lymphocytes, which was confirmed by Immunofluorescence double staining: 100% of FoxP3-positive cells were also CD3-positive und thus identified as Treg (Fig. 1D, E). To account for possible Treg subtypes described in the literature [22–24], we additionally performed double staining of FoxP3 and CCR4. Immunosuppressive effector regulatory T-cells (eTreg) specifically express the chemokine receptor in both blood and tumor tissue [23, 24]. We found that 70.6% of the FoxP3 + cells were also CCR4 + (Fig. 1F, G).

**Fig. 2:**
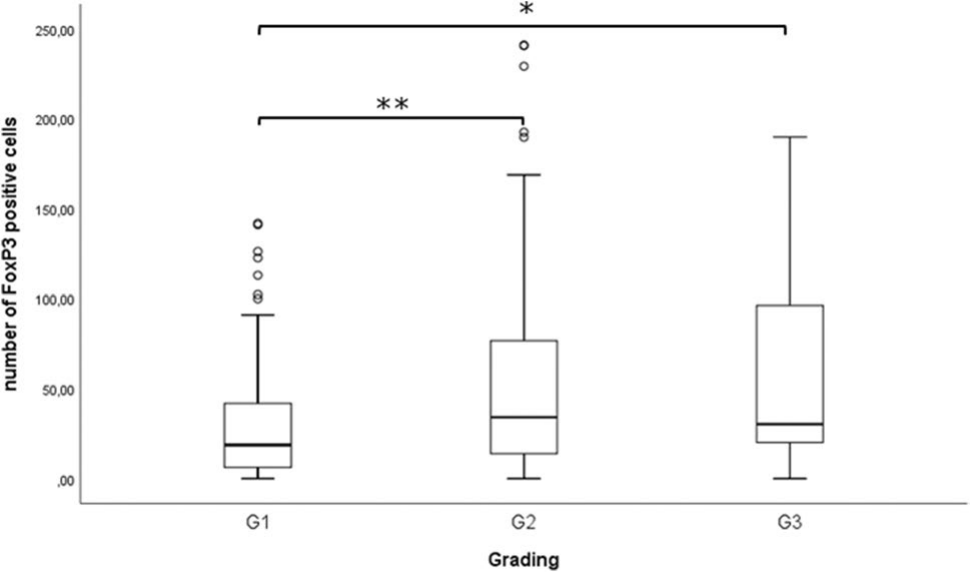
FoxP3+ cell infiltration increases with higher Grading in EC. A significant difference was found between G1 and G2, as well as G2 and G3. Mann-Whitney-U-Test. Boxplots display the five-number summary of data (minimum, first quartile, median, third quartile, maximum) based on the numbers of FoxP3+ celss/TMA spot. Isolated outliers over 250 were not shown for the sake of clarity

In the following analyses, the distribution of several clinic pathological parameters such as grading, T-status, N-status and FIGO-classification were examined. Regarding the grading, a significantly higher number of FoxP3 + cells was found in G2 (median 34.1) and G3 (median 30.3) compared to G1 (median 18.7). The Kruskal–Wallis-test revealed a significant difference between these three groups (*p* < 0.001) and Mann–Whitney-Test revealed a significant difference between G1 and G2 (*p* < 0.001) as well as G1 and G3 (*p* = 0.011), but not between G2 and G3 (Fig. 2, Table 2). Thus, enhanced numbers of FoxP3 + cells also correlated significantly with high grading (ρ = 0.239, *p* < 0.001). No further correlations to other clinic-pathological parameters were found (Table 2).

**Table 2:** Comparison with clinico-pathological parameters showing a significant association of the number of FoxP3 + lymphocytes with tumor grading (shown in bold). Values are median numbers of positive cells per TMA spot

	Number of FoxP3 + lymphocytes	***p*-**value (Mann–Whitney-U-/Kruskall-Wallis-test)
Tumor size pT		0.429
pT1 pT2 pT3 pT4	24.4 31.1 30.7 20.7	
FIGO staging		0.148
I II III IV	24.9 28.6 30.7 10.6	
Grading		< 0.001***
G1 G2 G3	17.7 34.1 30.3	
Nodal status		0.644
pN0 pN1	27.8 24.3	
Metastases		0.132
pM0 pM1	24.6 7.8	
Age at diagnosis		0.649
< 65 > 65	24.3 28.1	

Concerning the FIGO and pT stages, low staged and high staged (FIGO I & IV, pT 1 & 4) specimens showed a reduced number of FoxP3 + cells compared to middle-staged (FIGO II & III, pT 2 & 3) specimens. However, no significant association was reached (Supplement 4, Table 2), as well as with N-Status and M-Status. The data could, however, only be evaluated to a limited extent, since few patients had a positive N- or M- status (Table 1).

### High FoxP3 expression is associated with a poorer overall survival

**Fig. 3:**
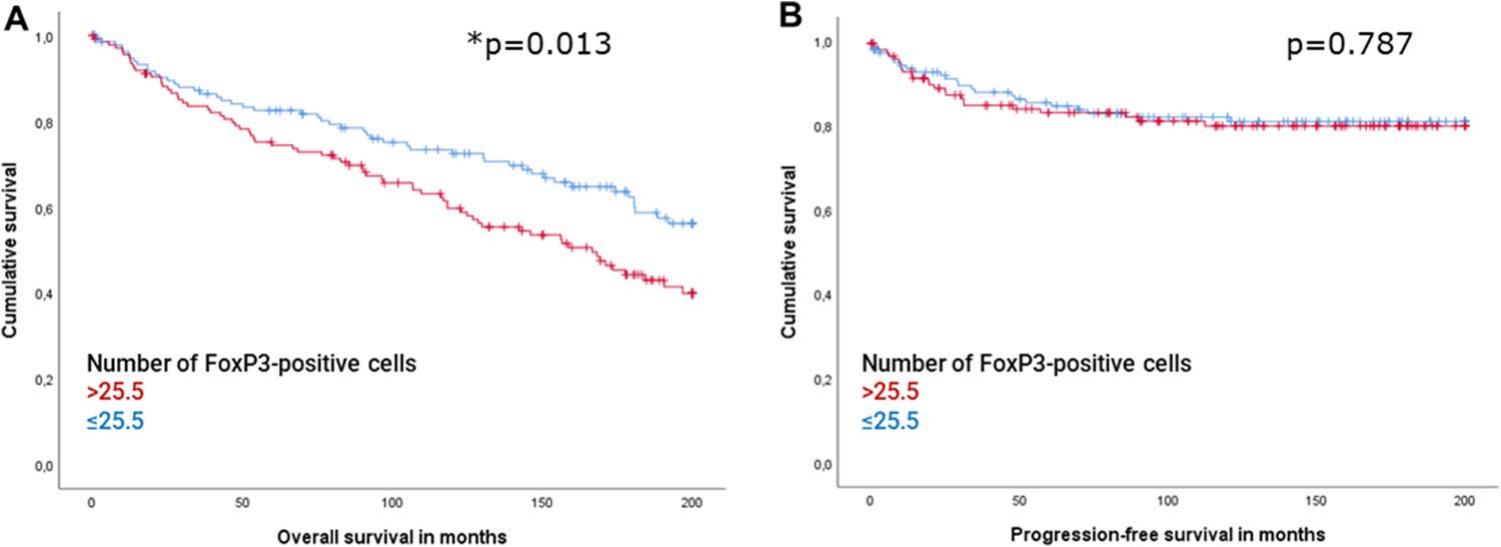
High FoxP3+ lymphocytic infiltration is associated with poor prognosis in EC. (**A**) Kaplan-Meier Curve showed a significantly shorter overall survival for high- grouped patiens (*p* = 0.013). (**B**) No difference in progression-free surival was found. The median number of FoxP3-positive lymphocytic cells per TMA-Spot was chosen as cut-off, that defined high-risk (>25.5) and low-risk groups (≤25.5). Results of the log-rank test are shown

The median number of FoxP3 positive cells (25.5) was chosen as cut-off to generate patient groups. A high number of FoxP3 + cells was significantly associated with a decreased overall survival (OS; *p* = 0.013, HR = 1.574, CI95% 1.095–2.261 in univariate regression; Fig. 3A). However, concerning the time of recurrence no difference was observed among patients with FoxP3 low or high (*p* = 0.787, Fig. 3B). The estimated 5-year probabilities for OS were 82.5% ± 3.3% (Mean ± Standard Error of the mean (SEM)) for high-grouped and 74.4% ± 3.8% for low-grouped patients. The American cancer society suggests a 5-year overall survival of 81% for EC patients [31], which is surpassed by the FoxP3 low group and clearly missed by the FoxP3 high group. Multivariate Cox regression model revealed that when adjusting for age, a strong presence of regulatory T-cells represented a 1.549-times increased risk of shortened OS during the study period (Table 3A, B). In contrast Treg were not able to explain variance in OS beyond the combined explanatory power of age, grading and FIGO or pT staging (Table 3C-E). Due to the high concordance, the FIGO and pT staging systems were included separately in the regression model. The results confirm comparable HR for OS in both cases.

**Table 3:** Multivariate Cox regression confirms independency of lymphocytic FoxP3 expression (A) as prognostic factor for OS when adjusting for age (B), but not with grading (C), pT (D) and FIGO-staging (E). Significant results are shown in bold

		p	Hazard Ratio (95%CI)
A	Covariate		
	FoxP3 high vs. low	0.014**	1.574 (1.095–2.261)
B	Covariate		
	FoxP3 high vs. low	0.018**	1.549 (1.078–2.226)
	Age at diagnosis	< 0.001***	1.081 (1.058–1.103)
C	Covariate		
	FoxP3 high vs. low	0.221	1.271 (0.865–1.868)
	Age at diagnosis	< 0.001***	1.079 (1.057–1.101)
	Grading (Reference G1)	0.001***	
	G1 vs. G2 G1 vs. G3	**0.045** ** < 0.001**	1.503 (1.009–2.238) 2.936 (1.635–5.272)
D	Covariate		
	FoxP3 high vs. low	0.509	1.138 (0.775–1.673)
	Age at diagnosis	< 0.001***	1.084 (1.062–1.107)
	Grading (Reference G1)	0.001***	
	G1 vs. G2 G1 vs. G3	0.294 **0.000**	1.249 (0.825–1.892) 3.102 (1.746–5.513)
	FIGO (Reference FIGO I)	< 0.001***	
	I vs. II I vs. III/IV	0.280 ** < 0.001**	1.473 (0.729–2.977) 2.904 (1.851–4.558)
E	Covariate		
	FoxP3 high vs. low	0.632	1.308 (0.894–1.915)
	Age at diagnosis	< 0.001***	1.083 (1.060–1.106)
	Grading (Reference G1)	0.001***	
	G1 vs. G2 G1 vs. G3	0.339 **0.000**	1.225 (0.808–1.855) 3.008 (1.699–5.326)
	pT (Reference pT1)	< 0.001***	
	1 vs. 2 1 vs. 3/4	0.169 ** < 0.001**	1.600 (0.819–3.127) 3.285 (2.042–5.286)

### Absence of FoxP3 positive cells in endometrial control tissue

**Fig. 4:**
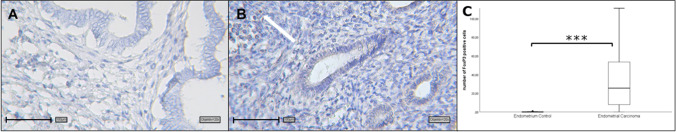
Representative images of Endometrium control group specimen with negative FoxP3 staining (**A**). In few cases a single regulatory T cell was found (**B**, white arrow). (**C**) Boxplot visualizes the significant (***) higher amount of Treg in EC than Endometrium control tissue. Boxplots display the five-number summary of data (minimum, first quartile, median, third quartile, maximum). Isolated outliers over 100 were shown for the sake of clarity. Scale bar 100μm, lens x20

Almost no FoxP3-expressing cells were found in the endometrium control group (Fig. 4A, B). The median number of FoxP3 positive lymphocytes per field of view (20x) normalised to the area was 0.00/mm^2^ (range 0–2). This result showed a significant difference to EC with a median number of 32.48/mm^2^ (*p* < 0,001; Fig. 4C).

### Increasing number of immunosuppressive Treg within PBMC after co-culture with EC cell lines

**Fig. 5:**
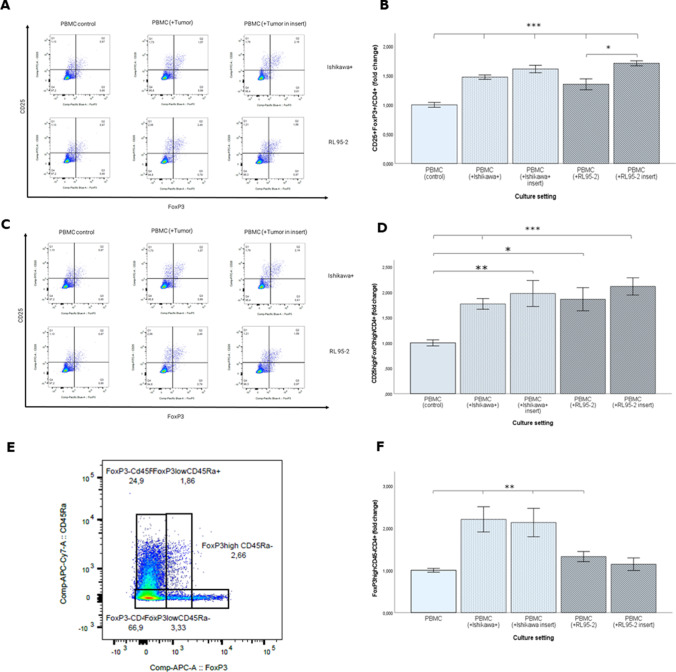
(**A**) Phenotype change of PBMC in transwell cultures after 72h. Percentage of CD25+FoxP3+/CD4+ cells in cultured PBMCs is determined by flow cytometry in direct and insert coculture with Ishikawa+ and RL95-2. PBMC cells cultured alone served as control. The horizontal axis and vertical axis represent the corresponding fluorescence phenotype expression of FoxP3 and CD25. First row represents PBMC coculture with Ishikawa+ cells and bottom row with RL95-2. (**B**) Histograms represent flow cytometry results showing increased of FoxP3 and CD25 in CD4+ population as fold change. Additional Gating was performed to separate the FoxP3hiCD25hi population (**C**) and results confirmed an increased percentage of eTreg after coculture (**D**). The additional Marker for immunosuppressive Treg CD45RA was introduced to strengthen a shift of Treg towards the immunosuppressive FoxP3highCD45RA-fraction after coculture with tumor cells (**E**, **F**) Error bars indicating ± SEM. Significant differences are marked with */***. All experiments are performed with two to three biological replicates

The effect of EC cells on the phenotype of T-cells was investigated in a co-culture system of Ishikawa + and RL95-2 cells and healthy donor PBMC. We evaluated the levels of CD25 + FoxP3 + Treg in the subgroup of CD4 + T-cells after co-culture, and found that the percentage of CD4 + CD25 + FoxP3 + Treg significantly increased after co-culturing for 72 h, in direct co-culture as well as in inserts compared to healthy donor PBMC cultured alone (Fig. 5A, B and Supplement 5). Absolute percentages are presented in Supplement 6 and 7, and confirm that the same applied when looking exclusively at the shift of the individual markers CD25 and FoxP3 (Supplement 7B, C). A significant increase of CD25^+^ cells could be detected in every group compared to PBMC control except from PBMC after direct co-culture with RL95-2. Differences between direct co-culture and insert setting were only found in combined CD25 + FoxP3-analysis in RL95-2 co-culture. Together a significant shift towards a higher percentage of CD25 + FoxP3 + cells was found in the CD4 + population, as well as after individual consideration of the two markers. According to Saito et al. [22], immunosuppressive Treg (eTreg) are characterised as CD25hiFoxP3high.Therefore, we extended the gating strategy including separation of high and low populations of each marker. The percentage of eTreg within PBMC in all co-culture settings was found to be significantly higher than in the control population of untreated PBMC. No difference was observed between co-culture in the insert or direct co-culture (Fig. 5C, D). Staining for CD45RA, whose absence is also described as characteristic for immuosuppressive Treg, revealed a statistically significant shift for PBMC in co-culture with both cell lines, but only a trend to increased Fraction III (FoxP3highCD45RA-) regarding RL95-2 in insert co-culture (Fig. 5E, F). This result further underlines our above CD25highFoxP3high-population analysis as being the most relevant marker combination to identify immunosuppressive Tregs.

**Fig. 6:**
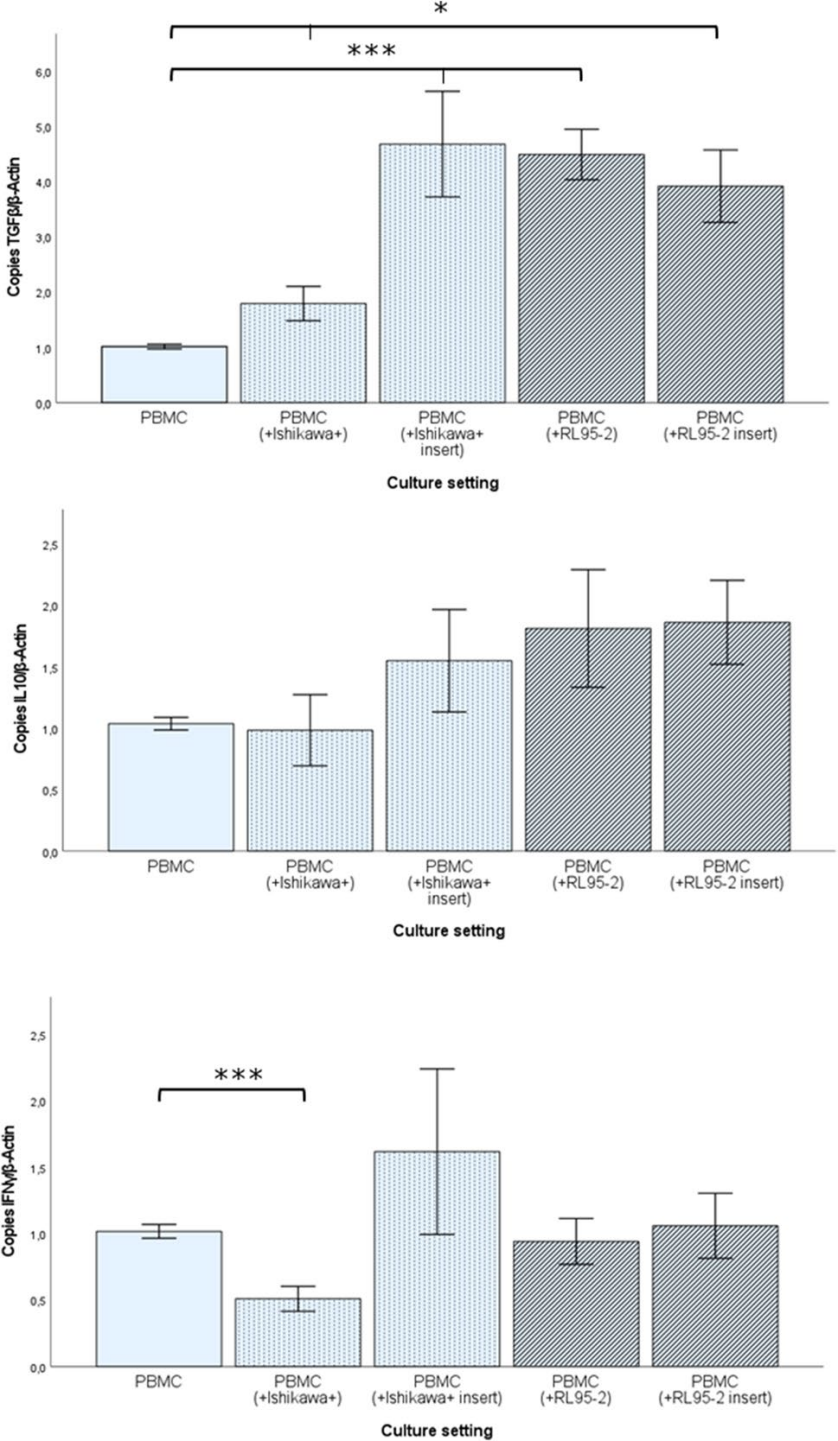
qPCR results of PBMC for pro- and anti-iflammatory cytokines after coculture with tumor cells. mRNA-levels of intiinflammatory cytokine TGFβ in PBMC increases statistically significant after coculture with tumor cells (**A**), while IL10 shows a trend to increased mRNA expression but fails to reach statistical significance (**B**). The level proinflammatory IFNγ remains stable except of an significant increase after direct coculture with Ishikawa+. Values presented as mean ± SEM. All experiments are performed in biological duplicates with the two housekeeping genes β-Actin

Additional sorting of the harvested PBMC in order to isolate the CD4 + fraction and subsequent RT-qPCR analysis of the immunosuppressive markers IL10 and TGFβ, as well as proinflammatory IFNγ, was performed to confirm the results from flow cytometry. We found that the TGFβ mRNA levels of CD4 + cells increased significantly in every co-culture setting compared to the PBMC control population (Fig. 6A), while IL10 showed a trend towards higher levels, but without statistical significance (Fig. 6B). To exclude a general upregulation of cytokine mRNA in the cells, also proinflammatory IFNγ was analysed. We found stable levels after co-culture, and in PBMC + Ishikawa + direct co-culture even a decreased level (Fig. 6C). To assess the impact of regulatory T-cells on tumor cells, functional assays were performed after co-culture of two EC cell lines with isolated Treg.

### Increased viability as well as invasive and migrative abilities of tumor cells after co-culture with Treg

**Fig. 7:**
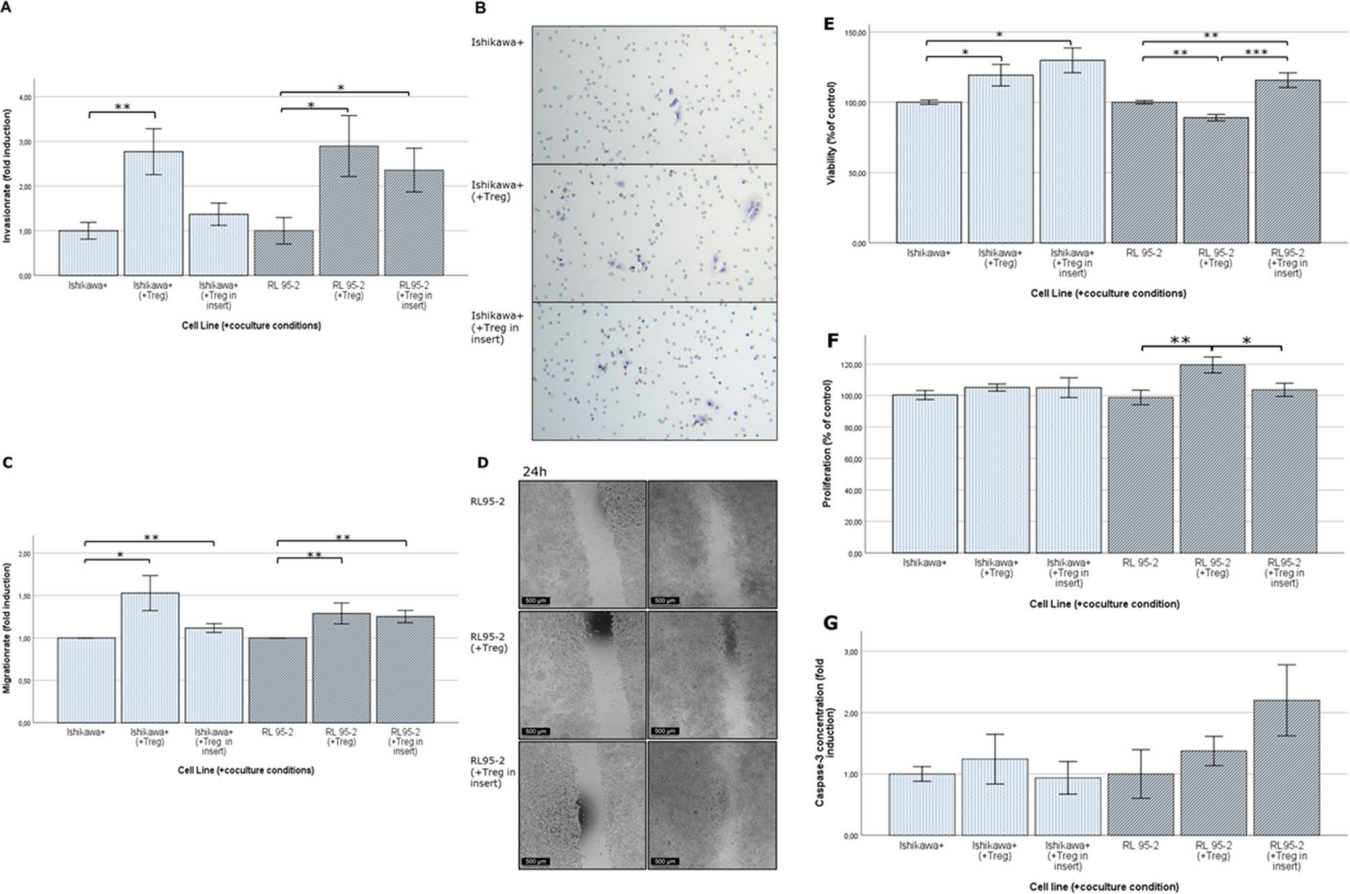
Presence of Treg favors cancer progression by an activation of viability, invasion and migration, while differences in proliferation- and apoptosisrate are marginal (**A**) Results of the invasion assay reveal, that invasion ability of tumor cells increases in the coculture system. (**B**) Representative images of Ishikawa cells after membrane invasion in different culture settings, lens 20x (**C**) The wound healing assay shows that also the migration ability of cells is significantly activated in direct and insert coculture compared to the tumor cells cultured alone (**D**) Representative images of wound healing assay with RL95-2 cells after 24 and 72h (**E**) Viability of changes after 72 hours coculture with sorted-CD4+CD25highCD127low Tregs. Only RL95-2 cells after direct coculture showed less viability compared to control. (**F**) Incorporation of BrdU showed only significant differences in RL95-2 cell line. (**G**) Caspase-3-concentrations were extremely low in each coculture conditions. Error bars indicating mean ± SEM. Significant differences are marked with */***. All experiments are performed with three biological and three technical replicates

Using a MTT assay, we found that the viability of Ishikawa + cells was significantly increased in direct co-culture with isolated CD4 + CD25 + CD127low Treg (*p* = 0.047) and even more in insert co-culture (*p* = 0.042) compared to the control group (Supplement 8; Fig. 7E). The same applied to RL95-2 cells in indirect co-culture (*p* = 0.008), while their viability was significantly decreased after direct co-culture (*p* = 0.002). Consequently, also a significant difference between direct and insert co-cultures was found (*p* < 0.001).

Next, the effect of Treg on the invasion potential of investigated tumor cell lines was tested in vitro in a Matrigel-coated membrane system. A significant invasive ability of EC cells could be observed after co-culturing Treg and tumor cells indirectly. In direct co-culture a significant difference could be found in both EC cell lines (Ishikawa + cells (*p* = 0.002) and RL-95–2 (*p* = 0.02)). In indirect co-culture a significant change in tumor invasion occurred only in RL95-2 cells (*p* = 0.047). Remarkably, the invasive ability was stronger in both cell lines after direct co-culture than after indirect co-culture (Fig. 7A, B; Supplement 8).

Tumor cell migration was attenuated by scratch wound healing assay. We found that the migration was significantly activated in insert and direct co-culture settings compared to the control group (Fig. 7C, D Ishikawa + /Treg *p* = 0.04, Ishikawa + /Treg insert *p* = 0.002, RL95-2/Treg *p* = 0.007, RL95-2/Treg insert *p* = 0.002; Supplement 8). These results indicate that the presence of regulatory T-cells promotes cancer progression.

### Marginal differences in proliferation and apoptosis of tumour cells after co-culture with Treg

Using a BrdU cell proliferation assay we found only differences in RL95-2 cells following co-culture with Treg (Fig. 7F; Supplement 8). An increased proliferation rate was detected in a direct co-culture setting in comparison to insert (indirect) co-culture (*p* = 0.027) as well as to control tumor cells cultured alone (*p* = 0.009). No effect was found for Ishikawa + cells. In addition, a Caspase-3 assay was performed to investigate apoptosis rates. We found that the overall Caspase-3 expression rates were very low and no significant differences were found in the different culture groups (Fig. 7G; Supplement 8). Due to the extremely low Caspase-3 activity (40–70 ng/ml) close to the detection limit of the ELISA, very high deviations resulted, indicating that EC cell apoptosis plays a minor role in the context of Treg co-culture.

## Discussion

In this study we examined the role of Treg in EC. The presence of FoxP3 positive cells in the TME were shown to correlate significantly with high grading and poorer overall survival rates. Co-culture of Ishikawa and RL95-2 cells with isolated Treg led to increased invasion and migration, but not to a significant difference in apoptosis. The features viability and proliferation were dependent on the cell line used.

FoxP3 + CD25 + CD4 + Treg play an important role in the process of immune tolerance. They are produced by the thymus and can be found in lymphoid and non-lymphoid tissues as well as in blood. Furthermore, they can also be detected in the tumor microenvironment (TME). In various cancer entities, a high number of Treg has been reported to be associated with a worse prognosis by suppressing anti-tumor responses [32–34] in, for example epithelial ovarian cancer [35, 36] and non-small-cell lung cancer [37]. It is well known, that the Treg marker FoxP3 can be upregulated or downregulated depending on the human cancer type [26, 38]. Sometimes it acts as a tumor promoter and sometimes as a tumor suppressor [38]. In EC FoxP3 expression in the TME seems to be associated with a poorer prognosis: a study with 200 EC patients showed that its expression correlated with poorer survival rates [39] and with mismatch repair-deficiency [40]. In our cohort, high numbers of FoxP3 positive lymphocytes led to significantly worse overall survival rates. This observation complies with the results of De Jong et al. who described Treg as a prognostic factor for progression free survival [41]. Several groups reported a correlation between grading, deep invasion and FoxP3 expression in EC [25, 41]. Our findings confirm the correlation between high grading (G3) and high numbers of FoxP3 positive cells in a large, representative cohort. The correlation of FoxP3 expression in the TME to TNM stadium remains, however, unclear [25, 42]. Giatromanolaki et al. showed a correlation between FoxP3 expression and a high vessel density, which may be linked to the observed poor prognosis, although they noted only a trend regarding survival rates [43]. This thesis was also supported by a mouse model: in breast cancer, Treg were found to play an important role in metastasis, while the cells could not metastasize in their absence [44, 45]. FoxP3 cannot only be expressed by lymphocytes, but also by tumor cells as has, for example, been observed in breast cancer [26]. As yet, it is not clear whether EC cells express FoxP3, and only few data exist [27]. We failed to detect any CD4-FoxP3 + cells, so we cannot support the idea of EC cells expressing FoxP3.

Using flow cytometry, we found that the amount of Treg in PBMC increased after co-culture with EC cells. This finding suggests that PBMC serve as a reservoir for Treg and that the differentiation of Treg can be stimulated by co-culture with tumor cells. This effect has also been observed in other tumor types like hepatocellular carcinoma, gastric cancer and non-small-cell lung cancer [37, 46–48]. Cao et al. additionally showed that (in hepatocellular carcinoma) the proliferation of CD4 + CD25- T-cells is inhibited [46]. The proliferation rate of Treg seemed to be even more increased when the tumor cells expressed Human Leucocyte Antigen G [47] or by transforming growth factor-ß1 [48]. In summary, these data indicate that induced Treg are immunosuppressive and promote tumor progression [35].

In order to specify the mechanism of Treg influencing the tumor’s behaviour, we performed co-culture experiments of isolated Treg with two EC cell lines, Ishikawa + and RL95-2. We found that this co-culture led to increased invasion, viability and migration of the tumor cells in direct as well as in indirect co-cultures. This goes along with the already described correlation to prognosis. Regarding proliferation, only RL95-2 cells reacted with a significantly higher proliferation rate in the direct co-culture compared to the indirect co-culture. This may be due to the fact that RL-95–2 is rather grading 2 compared to Ishikawa with grading 1. Regarding differences between direct and indirect co-cultures, it is well-known that there are significant differences regarding interleukins, cell–cell adhesion and tumor necrosis factor alpha levels, which can influence the behaviour of tumor cells [47]. Our data revealed that the invasion and migration abilities of EC cells are increased in both settings, but more striking in case of direct cell–cell contact. To detect apoptotic rates, we used a sensitive Caspase-3 ELISA method. As the Caspase-3 levels turned out to be very low, this assay failed to show any significant differences after co-culture of Treg and EC cells, although this method detects early stages of apoptosis. As viability, invasion and migration were increased after co-culture, we expected trends to decreased apoptotic rates. This has previously been observed in ovarian cancer cells, but also in that model no significant difference could be obtained [49].

Although the results of the functional in vitro assays support the results from immunohistochemical experiments, the effects could be even better highlighted through an increased purity of Treg isolated from PBMC. Although we could show that the amounts of Treg increase after co-culture of PBMC with EC, it is still necessary to improve our understanding of Treg recruitment. For this, it may be helpful to examine the chemokines CCL22 and CCL1 in the TME of EC, as has been reported for other cancer entities [50, 51]. A better understanding of the function of Treg and their interactions with cancer cells will help to address them as potential new targets in future EC therapies. Treg in the TME express mostly TIM3, which can be blocked by a monoclonal antibody. In a mouse model, this led to an increased anti-tumor response [52]. The immune-escape phenomenon could also be overcome by an anti-GITR-antibody in mice, while GITR was overexpressed in Treg [53]. These therapeutic options may complement other immunotherapies like ipilimumab and, thereby, improve the prognosis of advanced EC.

In conclusion, we showed that the presence of Treg in the TME is associated with a poorer prognosis in EC. Co-cultivation of Treg with EC cells led to an increased invasion, viability, migration and proliferation in EC cells, thereby providing a mechanistic background. A better understanding of the cytokines involved in this process will be necessary in order to identify starting points for targeting Treg as part of future therapy regimens in advanced EC.

## Supplementary Information

Below is the link to the electronic supplementary material.

Supplementary file1 (DOCX 1890 KB)

## Data Availability

All data generated or analysed during this study are included in this published article. For any questions, please contact M. Mannewitz: Mareike.Mannewitz@med.uni-muenchen.de.

## Code availability

Excel and SPSS 26.0 (SPSS, Inc., Chicago, IL, USA).

## Abbreviations

EC: Endometrial carcinoma
FoxP3: Forkhead boxprotein P3
OS: Overall survival
PBMC: Peripheral blood mononuclear cell
SEM: Standard error of mean
TME: Tumor microenvironment
Treg: Regulatory T-cell
