# Supplementary material for: Presence of regulatory T-cells in endometrial cancer predicts poorer overall survival and promotes progression of tumor cells
Source: Cell Oncol (Dordr). 2022 Sep 13;45(6):1171–85. doi: 10.1007/s13402-022-00708-2 (PMC9747805; doi:10.1007/s13402-022-00708-2)
Supplement: Supplementary file 1 — Supplementary file1 (DOCX 1890 KB) [file 13402_2022_708_MOESM1_ESM.docx]

**Supplements**

# **Presence of regulatory T-cells in endometrial cancer predicts poorer overall survival and promotes progression of tumor cells**


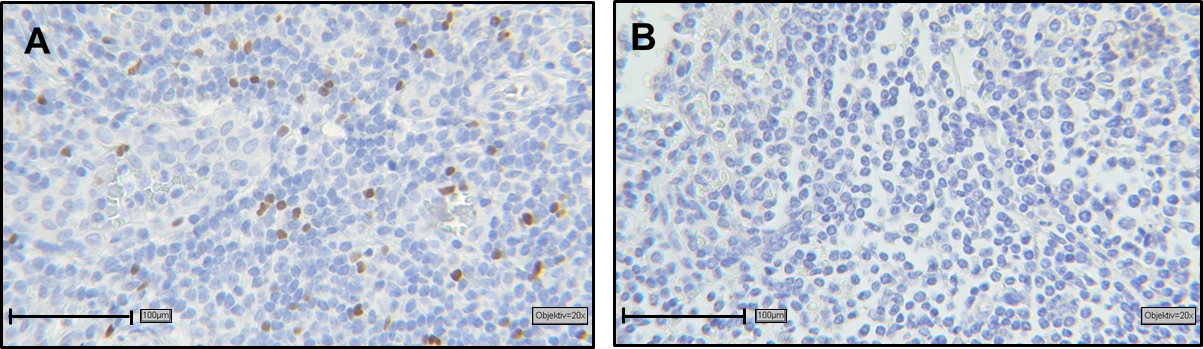


**Supplement 1.** Healthy tonsil tissue served as positive (**A**) and isotype (**B**) control for the IHC staining to determine the adequate dilution of the antibody and to detect potentially unspecific staining.


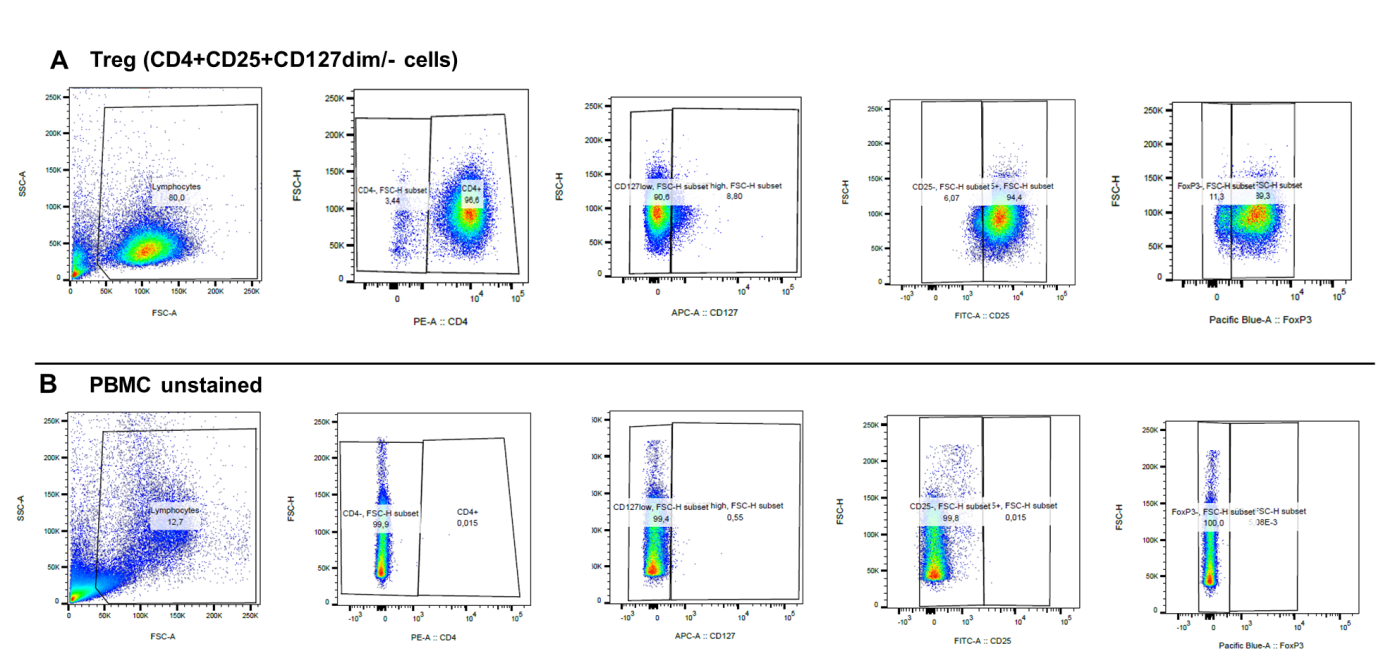


**Supplement 2.** Purity of isolated human regulatory T cells (CD4+CD25+CD127dim/‑ cells). Gating strategy was developed by analysing unstained PBMC (**B**) and then applied to the Treg-sample (**A**), obtained after MACS Isolation. Purity of isolated Treg is then expressed as percentage of CD4+CD25+CD127dimFoxP3+ cells in gated lymphocytes (74%). One additional analogue experiment confirmed this result (76% purity). Lymphocyte-gate was set in order to exclude dead cells, erythrocytes or thrombocytes.


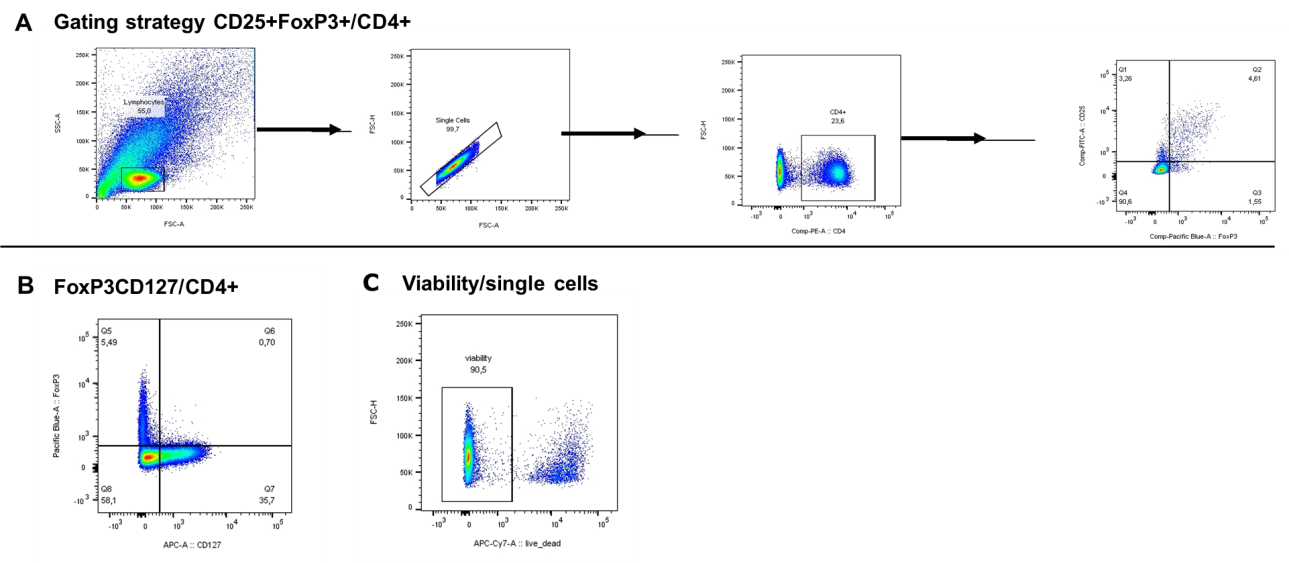


**Supplement 3.** Representative images for Gating strategy for CD25+FoxP3+/CD4+ cells and viability detection of harvested PBMC CD25+FoxP3+/CD4+ cells. (**A**) To detect proportion of CD25+ and/or FoxP3+ cells within CD4+ cells the following gating steps were performed: Lymphocyte – Single cells – CD4+ – CD25+/FoxP3+. (**B**) Additionally, CD127 was used as controlmarker, because Treg belong mainly to CD127low fraction. (**C**) Mean Viability of single cells was detected at 89,0% (Median: 90,5%, Range 73,2- 98,3%) with Zombie Viability dye (APC-Cy7).


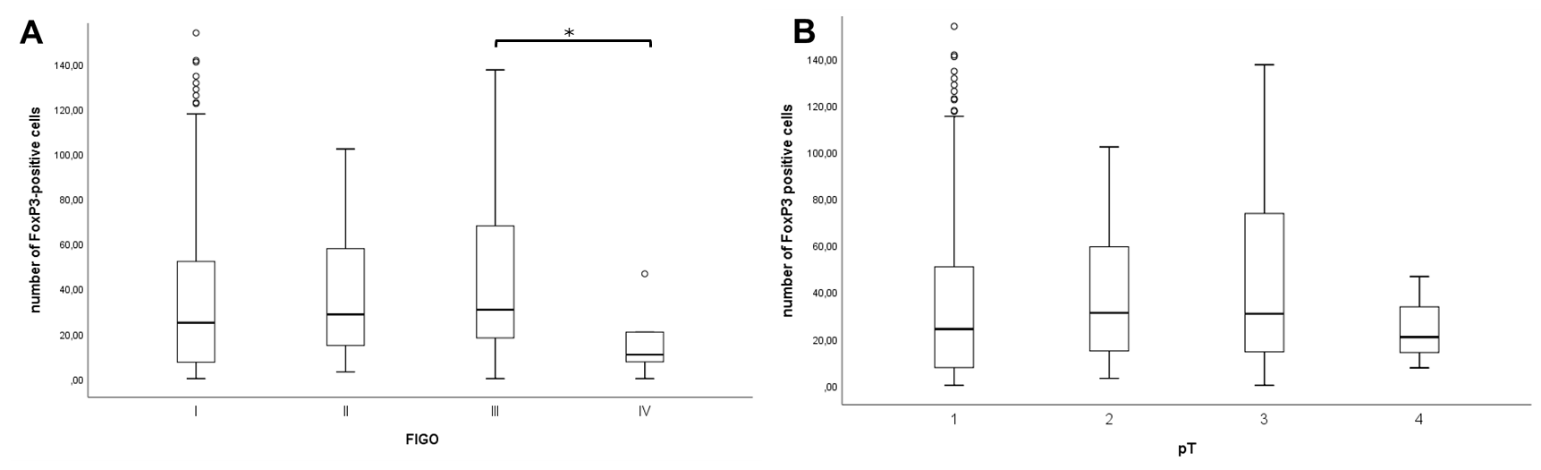


**Supplement 4.** Distribution of FoxP3-expression regarding FIGO (**A**) and pT-staging (**B**). High and low staged patients showed less expression than middle- staged (FIGO I/II, pT 2/3) patients. However, significance was only found between FIGO III and IV (p=0.044). Analysis was performed with Mann-Whitney-U test. Boxplots display the five-number summary of data (minimum, first quartile, median, third quartile, maximum) based on the numbers of FoxP3+ cells/TMA spot. Isolated outliers over 150 were not shown for the sake of clarity.

|  | **CD25+FoxP3+/CD4+** | **CD25+/CD4+** | **FoxP3+/CD4+** |
| --- | --- | --- | --- |
| **PBMC control** | 1,00 ±0,041 | 1,00 ±0,040 | 1,00 ±0,02 |
| **PBMC+ Ishikawa+** | 1,47 ±0,037 | 1,61 ±0,14 | 1,59 ±0,11 |
| **PBMC+ Ishikawa+ insert** | 1,61 ±0,064 | 1,51 ±0,17 | 1,72 ±0,16 |
| **PBMC+ RL95-2** | 1,35 ±0,093 | 1,41 ±0,19 | 1,48 ±0,15 |
| **PBMC+ RL95-2 insert** | 1,71 ±0,043 | 1,56 ±0,15 | 1,65 ±0,13 |

**Supplement 5.** Flow cytometry results revealed a shift within the PBMC concerning CD25 and FoxP3 after co-culture with tumor cells. Data are presented as fold induction to control PBMC. Values are Mean ± SEM.

|  | **CD25+FoxP3+/CD4+** | **CD25+/CD4+** | **FoxP3+/CD4+** |
| --- | --- | --- | --- |
| **PBMC control** | 2,91 ±0,22 | 4,605 ±0,32 | 3,79 ±0,26 |
| **PBMC+ Ishikawa+** | 4,07 ±0,22 | 6,24 ±0,61 | 5,25 ±0,30 |
| **PBMC+ Ishikawa+ insert** | 4,20 ±0,19 | 5,59 ±0,29 | 5,55 ±0,34 |
| **PBMC+ RL95-2** | 3,74 ±0,12 | 5,01 ±0,16 | 4,72 ±0,17 |
| **PBMC+ RL95-2 insert** | 4,43 ±0,26 | 5,86 ±0,37 | 5,34 ±0,25 |

**Supplement 6.** Percentage of CD25+ and/or FoxP3+ cells gated in total CD4+. Values are Mean±SEM.


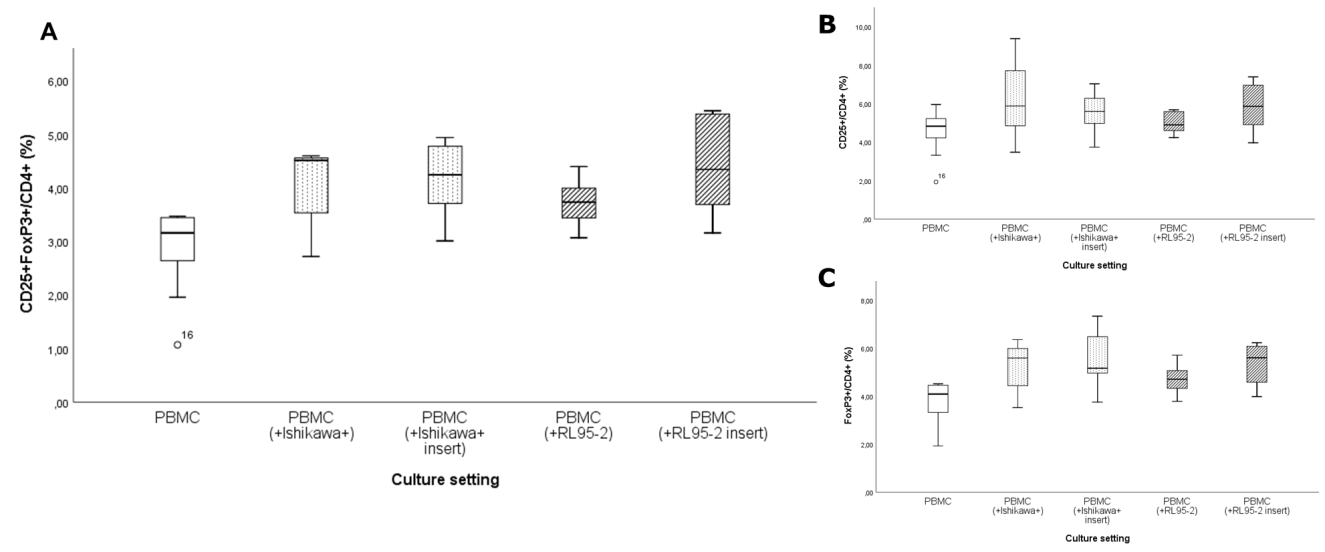
**Supplement 7.** Presentation of absolute percentages of CD25+FoxP3+/CD4+ cells in cultured PBMCs. Results led to fold change calculation in Figure 8 B-D. Boxplots display the five-number summary of data (minimum, first quartile, median, third quartile, maximum).

|  | **Ishikawa+ (control)** | **Ishikawa+**  **+ PBMC** | **Ishikawa+**  **+ PBMC insert** | **RL95-2 (control)** | **RL95-2**  **+ PBMC** | **RL95-2**  **+ PBMC insert** |
| --- | --- | --- | --- | --- | --- | --- |
| **Viability**  (MTT- Assay) | 1,000 ± 0,015 | 1,193 ± 0,076 | 1,298 ± 0,087 | 1,00 ± 0,011 | 0,891 ± 0,023 | 1,157 ± 0,051 |
| **Proliferation**  (BrdU-Assay) | 1,000 ± 0,025 | 1,032 ± 0,040 | 1,038 ± 0,032 | 1,000 ± 0,032 | 1,174 ± 0,105 | 1,050 ± 0,056 |
| **Invasion** (Transwellinvasion) | 1,000 ± 0,188 | 2,770 ± 0,513 | 1,369 ± 0,249 | 1,000 ± 0,296 | 2,896 ± 0,683 | 2,358 ± 0,489 |
| **Migration**  (Wound healing -Assay) | 1,000 ± 0,000 | 1,529 ± 0,207 | 1,117 ± 0,052 | 1,000 ± 0,000 | 1,290 ± 0,123 | 1,252 ± 0,073 |
| **Apoptosis**  (Caspase – Elisa) | 1,000 ± 0,120 | 1,242 ± 0,405 | 0,937 ± 0,266 | 1,000 ± 0,396 | 1,374 ± 0,239 | 2,200 ± 0,580 |

**Supplement 8**. Overview: Results of the functional assays of tumor cells after co-culture with PBMC. Values are mean ± SEM of fold induction relative to control (tumor cells only).
